# *HNF1A *gene polymorphisms and cardiovascular risk factors in individuals with late-onset autosomal dominant diabetes: a cross-sectional study

**DOI:** 10.1186/1475-2840-8-28

**Published:** 2009-06-02

**Authors:** Fernando MA Giuffrida, Gilberto K Furuzawa, Teresa S Kasamatsu, Marcos M Oliveira, Andre F Reis, Sergio A Dib

**Affiliations:** 1 Universidade Federal de São Paulo, Departamento de Medicina, Disciplina de Endocrinologia, R. Pedro de Toledo, 981 12o andar, Vila Clementino, Sao Paulo, SP – Brazil

## Abstract

**Background:**

Type 2 diabetes mellitus (T2DM) is a genetically heterogeneous disease, hepatocyte nuclear factor-1 homeobox A (*HNF1A*) single-nucleotide polymorphisms (SNPs) playing a minor role in its pathogenesis. *HNF1A *is a frequent cause of monogenic diabetes, albeit with early-onset. Some uncommon subgroups like late-onset autosomal dominant diabetes mellitus (LOADDM) may present peculiar inheritance patterns with a stronger familial component. This study aims to investigate the relationship of *HNF1A *SNPs with cardiovascular risk factors in this group, as well as to characterize them in contrast with classical T2DM (CT2DM).

**Methods:**

eighteen LOADDM (age at onset > 40 y.o.; diabetes in 3 contiguous generations, uniparental lineage) along with 48 CT2DM patients and 42 normoglycemic controls (N group) have been evaluated for cardiovascular risk factors and SNPs of *HNF1A*.

**Results:**

LOADDM showed significantly higher frequencies of SNPs A98V (22.2% vs 2.1%, p = 0.02) and S487N (72.2% vs 43.8%, p = 0.049) of *HNF1A *compared to CT2DM. I27L did not show significant difference (66.7% vs 45.8%), but associated with lower risk of hypertriglyceridemia (OR 0.16, 95% CI 0.04–0.65, p = 0.01). "Protective effect" was independent from other well-known predictive risk factors for hypertriglyceridemia, such as waist circumference (OR 1.09 per 1 cm increase, p = 0.01) and HDL (OR 0.01 per 1 mmol/l, p = 0.005), after logistic regression.

**Conclusion:**

Late onset autosomal dominant diabetes mellitus is clinically indistinguishable from classical type 2 diabetes individuals. However, LOADDM group is enriched for common *HNF1A *polymorphisms A98V and S487N. I27L showed "protective effect" upon hypertriglyceridemia in this sample of individuals, suggesting a role for *HNF1A *on diabetic individuals' lipid profile. These data contribute to the understanding of the complex interactions between genes, hyperglycemia and cardiovascular risk factors development in type 2 diabetes mellitus.

## Background

Type 2 diabetes mellitus (T2DM) is a clinically and genetically heterogeneous disease. The spectrum of T2DM ranges from lean and predominantly insulin-deficient individuals, to those more obese and insulin resistant [1]. Associated clinical conditions such as dyslipidemia, hypertension and visceral obesity, components of the Metabolic Syndrome (MS), further contribute to this heterogeneity.

Important advancements in the understanding of the genetic bases of T2DM have arisen in the last few years and several genes have now been associated to this disease. However, complete knowledge about the interaction among involved genes is still lacking. Curiously, most T2DM genes with previously known function are associated to insulin deficiency rather than insulin resistance (IR), as believed in the past decades [2].

MODY is a group of monogenic forms of diabetes, characterized by early onset and autosomal dominant inheritance, caused by mutations in 6 known genes. The most common are glucokinase (*GCK*) and hepatocyte nuclear factor-1 homeobox A (*HNF1A*), responsible for MODY2 and MODY3, respectively [3]. Effect of variants in these genes (or in their corresponding regions) upon T2DM has been demonstrated in genome-wide association studies [4,5]. This association is of small magnitude, as in most other T2DM genes. Families with clinical features of MODY but without mutations detected in any of the six known genes (termed MODYX) may phenotypically overlap with T2DM. MODYX individuals show greater age at diagnosis of diabetes than MODY and IR features such as obesity and dyslipidemia [6]. MODYX has been shown to be genetically heterogeneous, familial clustering of diabetes occurring either due to several unknown monogenic forms or by chance in a polygenic setting [7].

The involvement of *HNF1A *polymorphisms with MS has already been investigated in normal individuals [8-11], namely the Isoleucine to Leucine substitution on codon 27, located in exon 1, but with conflicting results. One study with a sample of Japanese individuals demonstrated a protective effect, with progressive higher levels of HDL-cholesterol with one and two Leucine alleles when compared to Isoleucine homozygotes [11]. Other study showed beta-cell function diminution which was progressive according to the number of *Leu *copies [10], and another demonstrated heterozygotes to have higher insulin sensitivity than both *Ile *and *Leu *homozygotes, albeit in an ethnically heterogeneous sample [9]. However, if the relationship of this variant with MS is still present in diabetes mellitus has not been as thoroughly investigated. Besides, the majority of studies approaching prevalence of this and other variants have been done in European and Japanese populations [12-19]. Brazilian population is one of the most heterogeneous in the world, with intense admixture among European, African and native Brazilian populations [20]. Moreover, MODYX appears to be more prevalent in Brazil than in European populations [21,22].

A more homogeneous group of type 2 diabetic individuals, as one with familial clustering, would be adequate to perform these studies in a heterogeneous population like this. Some uncommon T2DM families may show a strict autosomal dominant clinical pattern of inheritance, but with late onset typical of classical T2DM. Despite various reports studying familial clustering models as trios and multiplex families [23,24], this specific model has not been studied to our knowledge. Although the possibility that MODY mutations have a causal role in this group is remote, the exclusion of the most common MODY mutations by sequencing the whole *HNF1A *and *GCK *genes is necessary given the peculiar inheritance and onset patterns. A possible role for unknown variants in these individuals has also to be considered. These altogether grant investigation of characteristics that could individualize approach to this group regarding T2DM treatment. Since previous studies linked *HNF1A *variants to MS in normal individuals, investigating if this relationship persists in T2DM is appropriate.

This study aims to verify the frequency of exonic *HNF1A *variants in Brazilian individuals with late-onset autosomal dominant diabetes mellitus and classical T2DM, as well as to characterize its relationship to cardiovascular risk factors.

## Methods

Individuals with autosomal dominant (3 contiguous generations of familial history and uniparental lineage) and late-onset diabetes (age > 40 years old) have been studied, being arbitrarily denominated late-onset autosomal dominant diabetes mellitus (LOADDM) group. To differentiate them form classical MODY, late onset had to be present in all diabetic members of the family. Approximately 500 individuals from the Outpatient Diabetes Clinic at the Federal University of Sao Paulo have been screened for the inclusion criteria, eighteen individuals from 15 families meeting the criteria for the LOADDM group. Forty-eight unrelated individuals with classical T2DM (late-onset, any kind of familial history except either the one defining LOADDM group or both parents with diabetes) have been included as controls and denominated CT2DM group. All diabetic patients were included only if HbA_1c _values were below 8%, to avoid influence of severe beta cell dysfunction on the cardiovascular risk factors evaluated. Forty-two normoglycemic controls (N group) have been studied as well. Inclusion criteria for the N group were fasting blood glucose below 5.6 mmol/l and absence of diabetes in first-degree relatives. The study has been approved by the Ethics Committee of the Federal University of Sao Paulo, and informed written consent was obtained from all participants.

### Cardiovascular Risk Factors

Patients underwent complete physical examination and anthropometric evaluation. Blood pressure (BP) was measured with a mercury sphygmomanometer. Weight was measured with a clinical scale and stature with a stadiometer. BMI was calculated by dividing weight in kg by the square of height in meters. Waist circumference was measured at the midpoint between the rib cage and the iliac crest, determined along the median axillary line. Obesity was defined as a BMI ≥ 30 kg/m^2 ^and overweight as BMI ≥ 25 kg/m^2 ^(including those above 30 kg/m^2^). Metabolic syndrome components were evaluated by the NCEP-ATPIII criterion, two or more of them being diagnostic in diabetic patients (or three in the N group) [25].

### Laboratory methods

After a 12-hour fast, three blood samples with a 5-minute interval between each of them have been drawn, for C-peptide determination. Fasting blood glucose (FBG), HbA_1c_, total cholesterol, HDL-cholesterol, triglycerides, and anti-glutamic acid decarboxylase antibodies (GADA) have been analyzed in one of the samples. LDL-cholesterol has been calculated by Friedewald's equation. The following laboratory methods have been employed:

#### -Biochemistry

glucose-oxidase method has been used for FBG, colorimetric method for lipid profile and alkaline picrate for creatinine, using Bayer ADVIA 1650 automatic analyzer (Bayer Corp, Tarrytown, NY, USA). HbA_1c _was measured by HPLC (normal value: 4–6%).

#### -C-peptide

IFMA (autoDELFIA, Turku, Finland). Normal range (NR) 1.19–18.9 pmol/L and sensitivity 0.156 pmol/L pmol/L.

#### -GADA

radio-immunoassay (RSR Limited, Cardiff, UK) with 125-iodine-marked human recombinant GAD. Intra-assay variation coefficient 3.6% (4.2 U/mL); 2.9% (27.2 U/mL). Inter-assay variation coefficient 3.6% (4.2 U/mL); 5.1% (25.0 U/mL). Normal cut-off value 1.72 U/mL, corresponding to the 99th percentile of a sample of 194 normal individuals assessed at the Federal University of São Paulo Endocrinology Laboratory [26].

### Genetic analysis

Leukocyte DNA has been extracted from whole blood collected in EDTA tubes, employing commercially available kits (Gentra Systems, Minneapolis, MN, USA). In the LOADDM group, all exons and promoters of *HNF1A *and *GCK *have been amplified by PCR, utilizing Promega PCR Mastermix (Promega Corporation, Madison, WI, USA) and previously described primers [15]. PCR was performed according to previously described conditions [27]. Amplicons have been directly sequenced using BigDye 3.1 Sequence Termination kit in ABI3100 Genetic Analyzer (Applied Bio Systems, Foster City, CA, USA). All exons containing non-synonymous single-nucleotide polymorphisms (SNPs) have been sequenced in both CT2DM and N individuals.

### Homeostasis model assessment (HOMA) calculations

HOMA-%B and HOMA-IR calculations have been performed only in individuals not taking insulin, using fasting blood glucose and C-peptide values, with HOMA Calculator version 2.2.1 computer software [28].

### Statistical analyses

Patients have been divided in three groups as stated above (LOADDM, CT2DM and N). One-way ANOVA with Bonferroni correction has been employed to compare continuous variables and χ^2 ^for categorical ones. Non-normally distributed data have been normalized by logarithmic transformation. Pearson's correlation was used for continuous variables within groups. Diabetic patients have also been divided in groups according to genetic variants encountered and analyzed by Student's T-test and χ^2^. Variables with significant differences in univariate analysis were entered in Forward Logistic Regression (LR) models with Wald statistics. Hosmer-Lemeshow test was used for goodness-of-fit. In this test, observed values are compared to expected values obtained by the model. The higher the value of p is (less significant), better the fit of the model. Statistical power of LR models has been calculated post-hoc. Values of continuous variables are expressed in mean ± SD (or median and interquartile interval when not normally distributed), and categorical ones in percentages. A value of *p *< 0.05 (two-tailed) was considered significant. All analyses have been carried out in SPSS 13.0 for Windows software (SPSS Inc., Chicago, IL, USA), except for post-hoc LR power calculations, performed on PASS 2008 software version 08.0.5 (NCSS LLC, Kaysville, UT, USA).

## Results

No mutations or polymorphisms of *GCK *have been encountered in our sample. No *HNF1A *mutations causative of MODY have been found, as well. Three common non-synonymous SNPs of *HNF1A *have been found, two of them with a significantly higher prevalence in LOADDM group compared to CT2DM, as shown on Table 1. All SNPs were in Hardy-Weinberg equilibrium. I27L and S487N were in linkage disequilibrium (LD), with r^2 ^= 0.64. Anthropometric features, along with cardiovascular risk factors and MS prevalence, are detailed in Table 2. There were no significant differences between LOADDM and CT2DM groups.

**Table 1:** Frequencies of *HNF1A *SNPs I27L (rs1169288), A98V (rs1800574) and S487N (rs2464196) in the studied groups.

	genotypes	CT2DM^1^	LOADDM^2^	N^3^	*p*^4^
n		48	18	42	
					
I27L (rs1169288)	II (%)	54.2	33.3	42.9	
	IL+LL (%)	45.8	66.7	57.1	NS
					
A98V (rs1800574)	AA (%)	97.9	77.8	92.9	
	AV+VV (%)	2.1	22.2	7.1	0.02
					
S487N (rs2464196)	SS (%)	56.2	27.8	35.7	
	SN+NN (%)	43.8	72.2	64.3	0.049

^1^classical type 2 diabetes mellitus, ^2^late-onset autosomal dominant diabetes mellitus, ^3^normoglycemic; ^4^two degrees-of-freedom significance

**Table 2:** Characteristics of studied groups

				*p*	
				
	CT2DM	LOADDM	N	N vs CT2DM	N vs LOADDM
n	48	18	42	NS	NS
age (years)	65.6 ± 7.1	67.4 ± 10.8	63.0 ± 11.2	NS	NS
age at diagnosis (years)	54.9 ± 8.4	54.7 ± 10.0	-	-	-
weight (kg)	73.2 ± 13.7	73.4 ± 15.3	61.4 ± 13.4	<0.01	<0.05
BMI (kg/m^2^)	28.7 ± 4.2	28.5 ± 4.3	25.3 ± 3.7	<0.01	<0.05
waist (cm)	98.7 ± 10.7	99.4 ± 13.0	86.3 ± 9.9	<0.01	<0.01
Systolic BP (mmHg)	125.3 ± 16.0	118.0 ± 13.6	130.1 ± 17.3	NS	<0.05
Diastolic BP (mmHg)	75.2 ± 7.6	72.6 ± 8.7	81.8 ± 9.0	<0.01	<0.01
FBG (mmol/l)	7.18 ± 1.69	6.41 ± 1.91	4.98 ± 0.33	<0.01	<0.01
A1c (%)	6.4 ± 2.4	7.4 ± 1.2	5.5 ± 0.6	0.07	<0.01
total cholesterol (mmol/l)	4.44 ± 0.87	4.56 ± 0.97	4.94 ± 1.04	<0.05	NS
HDL-cholesterol (mmol/l)	1.23 ± 0.35	1.18 ± 0.28	1.43 ± 0.43	<0.05	0.07
LDL-cholesterol (mmol/l)	2.35 ± 0.64	2.64 ± 0.86	2.96 ± 0.84	<0.01	NS
triglycerides (mmol/l)	1.45 (1.07–2.07)	1.36 (1.05–1.84)	1.14 (0.78–1.48)	<0.05	NS
HOMA-%B^1^	77.1 ± 42.7	80.5 ± 46.1	107.8 ± 40.0	<0.01	NS
HOMA-IR^1^	2.12 ± 1.28	2.43 ± 1.39	1.27 ± 0.64	<0.05	NS
fasting C-peptide (nmol/l)	0.78 ± 0.50	0.77 ± 0.56	0.57 ± 0.29	NS	NS
					
				*p*^2^	
				
females (%)	68.8	72.2	83.3	NS	
hypertension (%)	85.4	72.2	50	<0.01	
obesity (%)	31.3	22.2	11.9	NS	
overweight (%)	83.3	66.7	52.4	<0.01	
central obesity (%)	62.5	61.1	38.1	0.051	
low HDL (%)	63.8	50	34.1	<0.05	
hypertriglyceridemia (%)	38.8	33.3	17.7	NS	
dyslipidemia treatment (%)^3^	8.3	0	7.1	NS	
metabolic syndrome (%)	87.5	77.8	11.9	<0.01	
insulin treatment (%)^4^	14.9	37.5	-	0.054	
positive GADA (%)	4.9	0	2.6	NS	

^1^calculated in 39 individuals from group CT2DM, 14 from LOADDM, and 39 from N; ^2^Two *df *significance, except ^4^(one *df*); ^3^current or previous use of fibrates/statins; NS denotes *p*< 0.10

Diabetic patients (i.e., only those from LOADDM and T2DM groups) have been divided in two groups according to I27L genotype, utilizing a dominant model, that is, group II (containing only Isoleucine homozygous individuals) and group IL+LL (containing both heterozygous and homozygous carriers of the Leucine allele). Comparison between groups is illustrated in table 3. No differences were observed in age, age at diagnosis, weight, BMI, waist circumference, FBG, HbA_1c_, blood pressure and lipid profile (as continuous variables). There were no differences between groups divided according to S487N genotype (SS and SN+NN) for the same comparisons (data not shown). Dividing normal individuals in the same way, no differences have been observed between the two groups, for both SNPs.

**Table 3:** Characteristics of diabetic patients (grouped LOADDM and CT2DM individuals) according to I27L polymorphism genotype.

	II	IL+LL	*p*
females (%)	71.9	67.6	NS
hypertension (%)	90.3	76.5	NS
obesity (%)	25	33.3	NS
overweight (%)	75	84.8	NS
central obesity (%)	59.4	64.7	NS
low HDL (%)	64.5	55.9	NS
hypertriglyceridemia (%)	74.2	50	0.045
dyslipidemia treatment (%)^1^	28.1	26.5	NS
metabolic syndrome (%)	90.6	79.4	NS
insulin treatment (%)	22.6	23.5	NS
positive GADA (%)	3.6	3.6	NS

^1^current or previous use of fibrates/statins

Prevalence of hypertriglyceridemia was significantly lower in individuals with the I27L SNP as compared to those without. Despite II and IL+LL groups differed only in hypertriglyceridemia in univariate analysis, patients with hypertriglyceridemia (regardless of genotype) were different from those with normal triglyceride levels in the commonly expected aspects: longer time from diagnosis of diabetes; higher mean waist circumference and percentage of insulin treated patients; lower mean HDL levels (and also higher prevalence of low HDL). These variables along with presence of I27L were entered as independent variables in a forward LR model, with hypertriglyceridemia as dependent variable, aiming at achieving highest goodness-of-fit (g.o.f.) and statistical power greater than 80%. HDL has been entered either as a continuous or categorical variable. Besides waist circumference, other measurements of adiposity have been entered (weight, BMI, or corresponding categorical variables). Gender was also included, since HDL and waist circumference can be considered gender-specific. Results are expressed in Table 4. From the three models depicted with power > 80%, Model 1 showed the best fit.

**Table 4:** Logistic regression models testing association of hypertriglyceridemia and I27L in diabetic patients (grouped LOADDM and CT2DM individuals).

Dependent variable: hypertriglyceridemia					
Independent variable^1^	OR (exp B)	95% C.I.	*p*	*p *for g.o.f.^2^	power (%)
**MODEL 1**					
I27L	0.16	0.04–0.65	0.011	0.78	91.6
time from diagnosis (years)	1.23	1.04–1.46	0.013		
waist circumference (cm)	1.09	1.02–1.17	0.014		
HDL (mmol/l)	0.01	0.001–0.26	0.005		
(insulin use)					
(female gender)					
					
constant	0.02		0.260		
					
**MODEL 2**					
I27L	0.19	0.05–0.73	0.016	0.36	87.1
waist circumference (cm)	1.09	1.02–1.17	0.018		
time from diagnosis (years)	1.16	1.02–1.32	0.023		
low HDL	9.52	2.36–38.45	0.002		
(female gender)					
					
constant	0.02		0.255		
					
**MODEL 3**					
I27L	0.17	0.04–0.69	0.013	0.29	88.5
waist circumference (cm)	1.09	1.02–1.17	0.016		
time from diagnosis (years)	1.15	1.01–1.31	0.033		
low HDL	8.89	2.21–35.78	0.002		
(female gender)					
(insulin use)					
					
constant	0.00		0.008		

^1^variables between brackets have been included but did not enter the model upon analysis; ^2^goodness-of-fit

None of the variables entered in the regression models showed multicollinearity among themselves, although time from diagnosis was longer in individuals taking insulin. Variables such as age, HbA_1c_, presence of hypertension, use of lipid medication, and belonging to the LOADDM group did not influence association of hypertriglyceridemia to I27L (data not shown). Excluding LOADDM patients from analysis, similar findings resulted.

Although triglyceride levels were not different between groups Ile and Leu as a continuous variable, they correlated moderately to adiposity measurements only in II (r 0.43, p = 0.02), but not in IL+LL (r 0.04, p = 0.84) group. Analogously, the same correlation occurred in CT2DM group (r 0.34, p = 0.02), but not in LOADDM (r -0.34, p = 0.20), and in SS group (r 0.53, p = 0.002), but not in SN+NN (r -0.15, p = 0.41) group.

## Discussion

Variants of *HNF1A *were associated to late-onset autosomal dominant diabetes mellitus (LOADDM) and modified the relationship between plasma triglyceride levels and waist circumference in Brazilian individuals with T2DM. The LOADDM group is enriched for common *HNF1A *polymorphisms A98V and S487N, although fitting within the heterogeneity of classical T2DM, being clinically indistinguishable from it. Furthermore, the presence of I27L variant in both LOADDM and CT2DM can show "protective effect" on the risk of hypertriglyceridemia afforded by its known predictive factors.

Higher frequencies of two SNPs not known to be in LD [4,18] were found in the LOADDM group, pointing to genetic differences between this group and classical T2DM. Besides, classical MODY has been excluded by complete sequencing of *HNF1A *and *GCK *genes. Nevertheless, in spite of I27L and S487N being in LD, we did not duplicate the same findings with both SNPs. A higher frequency of I27L in the LOADDM group would probably occur with an accordingly powered sample, but the rarity of individuals with this particular inheritance pattern precluded this task. However, studying pooled diabetic patients regarding presence of the I27L polymorphism, lower prevalence of hypertriglyceridemia in individuals with this SNP was seen. This finding has not been previously described. Protective effect of I27L on hypertriglyceridemia, with an OR in the order of 0.16, was significant in our sample even after correction for possible confounders. It occurred independently of other features already known to correlate with triglyceride levels such as time from diagnosis, waist circumference, and HDL [29]. Moreover, the first two variables showed suppressive effect upon I27L, which intensified statistical power of the association seen on univariate analysis.

Prevalence of I27L, A98V, and S487N in type 2 diabetic patients and normal controls has been widely studied in various European and Asian populations [12-19]. Overall, frequency of these three SNPs in our sample does not seem remarkably different from other populations, which fall mostly in the 40 to 50% range for I27L [10] and at the 3 to 7% range for A98V [12,18]. Data on prevalence of S487N are slightly underrepresented in literature, since it is commonly in strong LD with I27L, but their prevalence is similar [4,18]. Frequency of A98V and S487N in LOADDM group seems to deviate both from our controls groups and other populations. A similar finding has been reported only in a South Indian population, when a 20% or greater frequency of A98V occurred in individuals both with autosomal inheritance pattern and without familial history, albeit in individuals with early-onset type 2 diabetes. The pathophysiology of this specific T2DM subtype is poorly understood [30].

Our findings for I27L and hypertriglyceridemia are analogous to data previously reported for HDL [11], which is in accordance to a known pathophysiological connection [28] between both lipid subtypes. Among the different cardiovascular risk factors with a well-studied association to I27L [9-11], none of them occurred consistently, however they showed a common trend of protective effect upon MS and IR associated traits in non-diabetic individuals. These effects have not been similarly demonstrated in diabetic patients, but a subsequent publication showing subjects from the Botnia study to be more insulin-sensitive in presence of I27L [18] supports this hypothesis. The suppressive effect of adiposity measurements upon association of I27L and hypertriglyceridemia seen in our sample complements previous findings showing waist-to-hip ratio to act as a covariate on the association between I27L and IR [9]. Higher HDL levels are also associated to other variants of *HNF1A*, such as the G319S polymorphism private of the Oji-Cree population [31], reinforcing the role of *HNF1A *on lipid abnormalities associated to the MS. Interestingly, although I27L association with T2DM has been approached by several large sample studies [4,5], association with other MS components has not been correspondingly addressed.

Higher frequencies of A98V and S487N in LOADDM group are probably a specific feature of this subset of type 2 diabetic patients, since CT2DM and N groups showed frequencies similar to other populations, but might also stem from differences among them. Given the scarcity of these families, this could possibly occur in other ethnic groups but would be diluted in the whole T2DM population. Absence of *GCK *variants in our sample is not surprising, since there are no exonic variants as common as those encountered in *HNF1A *[32].

Since the majority of previous data about I27L and MS traits has been drawn from Asian and European samples, differences among studied populations are a possible explanation for the association of I27L and hypertriglyceridemia in our study. Since I27L is very rare in African populations [33], but Brazilian individuals described as Caucasians might carry up to 28% of African ancestry markers [20], this variant could be modulated by a different set of genetic traits in European and Brazilian populations.

Suppressive effect of adiposity measurements upon "protective" effect of I27L on hypertriglyceridemia raises the hypothesis that I27L could be preventing central obesity from increasing IR, although mechanisms linking I27L and IR are largely unknown [18]. The expected positive correlation between triglycerides and measurements of adiposity [34] occurred in patients without I27L and S487N polymorphisms, and analogously in the CT2DM group. Conversely, correlations were absent in individuals carrying the SNPs and also in LOADDM group. These facts altogether support this view, but at the same time raise the question whether the contribution of I27L to hypertriglyceridemia, despite being independent of other cardiovascular risk factors, is dependent on inheritance pattern. Autosomal dominant inheritance did not enter any of the LR models, and the same models applied only to CT2DM patients brought essentially the same results for hypertriglyceridemia. Besides, the fact that S487N was more prevalent in the LOADDM group, but did not associate with cardiovascular risk factors, may point to an independent role of I27L.

*HNF1A *mutated with variants I27L, A98V and S487N has been demonstrated to have decreased transcriptional activity upon genes involved in glucose metabolism (GLUT-2). This transcriptional impairment is situated midway between normal and MODY causative mutations [18]. Nevertheless, genes encoding other proteins involved in lipid metabolic pathways (such as 3-hydroxy-3-methylglutaryl coenzyme A reductase, for example) are also known targets for *HNF1A *[35]. Interaction of *HNF1A *with lipid hepatic metabolism in diabetes has been object of relatively little study, and mostly in MODY individuals [36]. In spite of I27L and S487N similar transcriptional impairment, the first is probably accountable for the described phenotypes, since it is located in the dimerization domain, a highly conserved region of the *HNF1A *gene, with greater functional significance [35]. An indirect effect of other SNPs such as S487N through LD, although not totally excluded, is unlikely.

## Conclusion

These data show a group of late-onset autosomal dominant diabetic individuals enriched for *HNF1A *polymorphisms, despite being clinically indistinguishable to classical T2DM. Concurrently, we report "protective" association of I27L, a common polymorphism of *HNF1A*, to hypertriglyceridemia in a sample of Brazilians with T2DM. This finding, along with other previously described evidence, point to a role of *HNF1A *in cardiovascular risk factors development in adult hyperglycemic individuals, which should be better understood through trans-ethnic investigation in future studies. Association studies linking this gene primarily to lipid phenotypes in diabetes are warranted. The LOADDM group is a potentially interesting model for genetic studies in T2DM, since it could possibly represent a concentration of diabetes polygenes in the same individuals in a degree greater than would be expected by chance alone.

## Abbreviations

BP: blood pressure; CT2DM: classical type 2 diabetes mellitus; FBG: fasting blood glucose; IR: insulin resistance; LD: linkage disequilibrium; LOADDM: late-onset autosomal dominant diabetes mellitus; LR: logistic regression; MS: metabolic syndrome; N: normoglycemic; SNP: single nucleotide polymorphism.

## Competing interests

The authors declare that they have no competing interests.

## Authors' contributions

FMAG carried out the molecular genetic studies, participated in the sequence alignment, participated in the design of the study, performed the statistical analysis and drafted the manuscript. GKF carried out the molecular genetic studies and participated in the sequence alignment. TSK carried out the immunoassays and molecular genetic studies. MMO participated in the selection and clinical evaluation of the patients. AFR and SAD conceived of the study, participated in its design, coordination and patient selection, and helped to draft the manuscript. All authors read and approved the final manuscript
